# Acute Retention of Urine—I

**Published:** 1908-07-04

**Authors:** 


### SURGERY.

### ACUTE RETENTION OF URINE—I.

Acute retention of urine is a grave surgical emergency, and as such is a condition whose treatment may demand great discrimination and skill. The condition is, of course, the outcome of a variety of causes, but by far the commonest of these is organic urethral stricture due to past gonorrhœa. In such a case the history is more or less typical.

The patient has had gonorrhœa, and this may have occurred a surprising number of years before. The writer can call to mind one patient who developed the symptoms of a stricture 45 years after an attack of gonorrhœa. It will be found that recently the patient has had increasing difficulty in passing his water, that the stream has become gradually smaller, and, finally, that he has to strain to start the act. And then on top of this syndrome he is suddenly quite unable to pass water at all. This the patient himself often attributes to a chill, but the commonest cause in such a case is that the patient has been indulging in alcohol. But why should alcohol produce acute retention? It is commonly said that the alcohol leads to congestion of the urethra, and thus completely occludes the already constricted passage. But such a statement is really a mere hypothesis. It seems as reasonable to suggest that the alcohol produces the same effect upon the unstriped muscle of the bladder as it does upon the voluntary muscle—that is to say, a condition of lassitude which prevents it from doing its work properly, so that the overworked detrusor muscles, which have already been exerted to their utmost to expel the urine, are now incapable of performing their duty. Or again, spasm may play some part. Whenever one is dealing with obstruction to the function of a tube in the body it is well to remember that the condition is more than half due to spasm, and less than half to organic obstruction.

The condition of the patient will vary according to the length of time which has elapsed since he has been able to pass his water. At first he suffers from inconvenience merely, but as the bladder begins to distend this becomes more and more acute until finally his condition is one of very severe suffering; in fact, there are few conditions which can produce such agony as retention of urine. On examination of such a case the bladder is found to reach up to or above the umbilicus, as can be discovered by percussion; but percussion must be performed tenderly, because any pressure on the hypogastrium increases the patient's discomfort. Where the condition has persisted for some time, urine may be dribbling away from the urethra; this is known as retention with overflow. This is a common cause of incontinence, and all patients whose water is dribbling away should be examined to see if the condition is due to a genuine incontinence or to an over-distended bladder.

There is a perplexing class of case in which no urine is passed by the patient, and he suffers the distress associated with this symptom, but on examination the bladder is found hardly to be distended at all, or if a catheter can be introduced only quite a small amount of urine is withdrawn. In these cases, which are not very rare, one must suppose that there has been a temporary suppression of the renal function secondary to the obstruction. But it is extremely valuable to know that such cases exist, or one might be led into thinking that the bladder was still distended even after the passage of a catheter and into aspirating suprapubically.

The other causes of retention of urine are far less common; it may arise from the impaction of a calculus or fragment of blood clot in the urethra. The former is one of the few causes of acute retention in the female, while the latter is generally associated with enlargement of the prostate, especially if the patient has been in the habit of passing a catheter for himself. The enlarged prostate bleeds with great readiness, and sooner or later the patient is sure to damage it, and this may result in the formation of a blood clot. The clot is swept along the urethra and is usually impacted not very far from the meatus, at a point opposite the base of the glans penis, where there is an anatomical narrowing of the urethra. The writer remembers such a case. The patient was an old man aged 79, who found himself suddenly unable to pass water one morning. He suffered from an enlarged prostate, and used to pass a catheter when he had any trouble with micturition. He tried to catheterise himself, but was unable to introduce the catheter more than an inch or so. He continued all day in increasing distress, and did not seek advice about his condition until the evening. On attempting to pass a catheter an obstruction was met opposite the base of the glans penis. In view of the history it was probable that it was an impacted blood clot, and firmer pressure was brought to bear; the clot was then dislodged and the urine at once began to flow. In this case the urine was drawn off by degrees, but even this precaution did not prevent him from suffering afterwards from more or less severe collapse and some hæmaturia.

It is a very necessary precaution to draw off the urine from a distended bladder very slowly, especially when the patient is an old man. Apart from the shock involved in emptying it suddenly, the relief of tension is so sudden that the capillaries in the bladder give way and troublesome hæmaturia may result. An enlarged prostate may give rise to retention of urine in other and more direct ways. The so-called middle lobe, for instance, which is, it is true, more often heard about than seen, may fall over the orifice of the urethra and entirely prevent the passage of urine, or as the result of instrumentation the whole organ may become so congested as to occlude the prostatic urethra.

Retention which is just as acute as the variety which arises from organic causes may result from tabes dorsalis, or from an injury to the detrusor centre in the spinal cord. The differential diagnosis, however, is an easy matter, since the passage of a catheter will set all doubt at rest; and a thorough general examination of the patient must then be made to establish the cause.

